# 4D cardiovascular magnetic resonance velocity mapping of alterations of right heart flow patterns and main pulmonary artery hemodynamics in tetralogy of Fallot

**DOI:** 10.1186/1532-429X-14-16

**Published:** 2012-02-07

**Authors:** Christopher J François, Shardha Srinivasan, Mark L Schiebler, Scott B Reeder, Eric Niespodzany, Benjamin R Landgraf, Oliver Wieben, Alex Frydrychowicz

**Affiliations:** 1 Departments of Radiology, University of Wisconsin - Madison, School of Medicine and Public Health, 600 Highland Avenue, Madison, WI 53562; 2 Departments of Pediatrics, University of Wisconsin - Madison, School of Medicine and Public Health, 600 Highland Avenue, Madison, WI 53562; 3 Departments of Medical Physics, University of Wisconsin - Madison, School of Medicine and Public Health, 600 Highland Avenue, Madison, WI 53562; 4 Departments of Biomedical Engineering, University of Wisconsin - Madison, School of Medicine and Public Health, 600 Highland Avenue, Madison, WI 53562; 5 Departments of Medicine, University of Wisconsin - Madison, School of Medicine and Public Health, 600 Highland Avenue, Madison, WI 53562; 6 Department of Radiology, University Hospital Schleswig-Holstein, Germany

## Abstract

**Background:**

To assess changes in right heart flow and pulmonary artery hemodynamics in patients with repaired Tetralogy of Fallot (rTOF) we used whole heart, four dimensional (4D) velocity mapping (VM) cardiovascular magnetic resonance (CMR).

**Methods:**

CMR studies were performed in 11 subjects with rTOF (5M/6F; 20.1 ± 12.4 years) and 10 normal volunteers (6M/4F; 34.2 ± 13.4 years) on clinical 1.5T and 3.0T MR scanners. 4D VM-CMR was performed using PC VIPR (Phase Contrast Vastly undersampled Isotropic Projection Reconstruction). Interactive streamline and particle trace visualizations of the superior and inferior vena cava (IVC and SVC, respectively), right atrium (RA), right ventricle (RV), and pulmonary artery (PA) were generated and reviewed by three experienced readers. Main PA net flow, retrograde flow, peak flow, time-to-peak flow, peak acceleration, resistance index and mean wall shear stress were quantified. Differences in flow patterns between the two groups were tested using Fisher's exact test. Differences in quantitative parameters were analyzed with the Kruskal-Wallis rank sum test.

**Results:**

4D VM-CMR was successfully performed in all volunteers and subjects with TOF. Right heart flow patterns in rTOF subjects were characterized by (a) greater SVC/IVC flow during diastole than systole, (b) increased vortical flow patterns in the RA and in the RV during diastole, and (c) increased helical or vortical flow features in the PA's. Differences in main PA retrograde flow, resistance index, peak flow, time-to-peak flow, peak acceleration and mean wall shear stress were statistically significant.

**Conclusions:**

Whole heart 4D VM-CMR with PC VIPR enables detection of both normal and abnormal right heart flow patterns, which may allow for comprehensive studies to evaluate interdependencies of post-surgically altered geometries and hemodynamics.

## Background

Tetralogy of Fallot (TOF) - consisting of ventricular septal defect (VSD), pulmonary stenosis (PS), over-riding aortic root, and right ventricular hypertrophy (RVH) - is one of the most frequent complex congenital heart defects, accounting for 9-14% of all congenital heart defects [1]. Since the development of more sophisticated and comprehensive cardiovascular surgical techniques over the last 20 years, long-term survival is expected. Therefore, the physiological sequelae of these surgical procedures, aimed at increasing flow to the pulmonary circulation, are often encountered in adolescents and adults. Following repair, patients with TOF frequently present with pulmonary regurgitation (PR) and/or residual or recurrent pulmonary artery stenosis (PS). Cardiovascular magnetic resonance (CMR) is routinely used to follow patients after TOF repair to monitor for changes in right ventricular (RV) size and function related to PR [2-5].

Four-dimensional (4D) flow-sensitive velocity mapping (VM) CMR [6] is increasingly being used for the visualization of complex flow patterns, which may prove helpful in determining functional outcomes following surgery for complex congenital heart disease [7]. In addition to providing insights into qualitative changes in flow patterns, 4D VM-CMR can also be used to quantify flow through any vessel of interest *a posteriori*, including in patients with TOF [8]

A limitation of 4D VM-CMR, especially when applying Cartesian encoding, is the compromise between length of acquisition, volume coverage, and spatial resolution. To keep scan times to tolerable durations, the imaging volume is typically limited to a moderately thick slab and spatial resolution has to be sacrificed. To overcome limitations of Cartesian 4D-VM CMR, radial undersampling provides large volume coverage with high spatial resolution in reasonable scan times. Radial acquisitions are also advantageous, relative to Cartesian acquisitions, because of their reduced susceptibility to motion artifacts [9]. The purpose of this study was to demonstrate the feasibility of implementing a true three-dimensional (3D) radially-undersampled 4D VM-CMR technique for the analysis of right heart flow patterns and quantification of pulmonary artery flow in normal volunteers and subjects with TOF.

## Methods

### Patients and Volunteers

This prospective study was approved by the Institutional Review Board (IRB) and was compliant with the Health Insurance Portability and Accountability Act. In accordance with our IRB protocol, written informed consent was obtained from all subjects ≥ 18 years of age; written informed consent from parents/legal guardians and subjects was obtained for subjects 15-17 years of age; written informed from parents/legal guardians and assent from subjects were obtained for subjects 7-14 years of age; and written informed consent from parents/legal guardians only was obtained for subjects ≤6 years of age. After obtaining appropriate subject consent, CMR studies were performed in 11 subjects (Table 1) with palliatively treated or repaired Tetralogy of Fallot (rTOF) - five men and six women; 20.1 ± 12.4 years; range: 7-43 years - and 10 normal volunteers without any known cardiovascular disease - six men and four women; 34.2 ± 13.4 years; range: 21-54 years. The difference in mean age between the two groups was statistically significant (Student's t-test, P < 0.05).

**Table 1:** Characteristics of Tetralogy of Fallot subjects

Subject	Gender	Age at time of CMR (years)	Age of last repair	Type of repair	Prior shunt
1	F	11	8 months	Transannular patch	Blalock-Taussig × 2
2	M	43	N/A		Waterston
3	F	15	2 years	Transannular patch	Blalock-Taussig
4	F	17	2 years	Transannular patch	N/A
5	F	7	2 months	Transannular patch	N/A
6	M	34	16 years		Waterston
7	F	13	6 months	Transannular patch	N/A
8	M	19	10 months	Transannular patch	N/A
9	M	9	6 months	Transannular patch and pulmonary valvectomy	Blalock-Taussig
10	M	38	N/A		Waterston
11	F	15	6 months	Homograft	N/A

N/A - not available or not applicable

CMR examinations in volunteers were all performed at 3.0T (MR750, GE Healthcare, Waukesha, WI). CMR examinations in rTOF subjects were acquired on clinical 1.5T (HDx, GE Healthcare, Waukesha, WI) and 3.0T (MR750, GE Healthcare, Waukesha, WI) CMR scanners. The choice of 1.5T or 3.0T was partially based on clinical availability of the scanners at the time of scheduling and the need for sedation. In the patients with rTOF, 4D velocity mapping was performed following the acquisition of clinically indicated CMR examinations. The clinical CMR protocol included axial 2D multislice bSSFP CINE acquisitions to quantify RV size and function as well as standard 2D PC to confirm or exclude the presence of PR and PS (Table 2). In healthy volunteers free of cardiovascular disease, only 4D velocity mapping was performed.

**Table 2:** Right ventricular and main pulmonary artery parameters in Tetralogy of Fallot subjects

Parameter	Mean ± standard deviation (range)
EF (%)	54 ± 7 (42-62)
EDV (mL)	185 ± 93 (39-378)
EDVI (mL/m^2^)	121 ± 48 (23-185)
ESV (mL)	87 ± 55 (19-203)
ESVI (mL/m^2^)	55 ± 26 (11-93)
PR (%) N = 8/11	37 ± 16 (5-56)

EF = ejection fraction; EDV = end-diastolic volume; EDVI = EDV index; ESV = end-systolic volume; ESVI = ESV index; PR = pulmonary regurgitant fraction.

### 4D velocity mapping CMR technique

PC VIPR was performed after intravenous (IV) administration of 0.1 mmol/kg gadobenate dimeglumine (Bracco Diagnostics, Princeton, NJ) in 8/11 subjects with rTOF and in 10/10 healthy volunteers. In subjects with TOF, IV contrast was given as part of the clinically indicated magnetic resonance angiography. IV contrast was not given to 3/11 subjects with rTOF due to the inability to obtain IV access (n = 2) and risk factors for nephrogenic systemic sclerosis (n = 1). A benefit of performing 4D VM-CMR after the administration of IV contrast is that it is known to improve signal-to-noise and velocity-to-noise performance [10].

4D VM-CMR was performed using PC VIPR, a previously described three-dimensional (3D) radially-undersampled, three-directionally velocity encoded PC technique [11]. To compensate for periodic motion due to breathing and cardiac motion, retrospective ECG-gating and respiratory gating with 50% trigger efficiency with an adaptive gating scheme based on bellows readings were applied, respectively. The resulting scan time varied individually and was on the order of 9-17 minutes. Sequence parameters were adapted to each individual's anatomy: field of view = 260-320 mm^3^; matrix = 256^3^; true spatial resolution = 1.02-1.25 mm^3 ^isotropic; velocity encoding = 40-400 cm/s, flip angle = 7-20°. TR/TE for acquisitions at 1.5T were 8.8-10.9/2.8-3.7 ms and at 3.0T were 6.2-6.7/2.0-2.2 ms. The nominal temporal resolution (4*TR) was, therefore, 35-44 ms at 1.5T and 25-27 ms at 3.0T.

### 4D velocity mapping data processing, visualization, and analysis

Automated off-line post-processing was applied to ensure that high quality images were reconstructed using several correction schemes that accounted for the effects of T_1_-saturation, *k-*space trajectory errors, motion, and aliasing associated with undersampling [12,13]. The number of reconstructed temporal frames was limited to 20 frames in order to keep file sizes at a manageable size. As part of the image reconstruction, a velocity-weighted MR angiogram was calculated from the PC VIPR dataset to facilitate orientation during visualization with the following software algorithms. Angiogram and noise masking were performed with a home-built MatLab-based software (The MathWorks, USA). Using the same tool, data was then converted for visualization with EnSight (CEI, Apex, NC).

**Figure 1:**
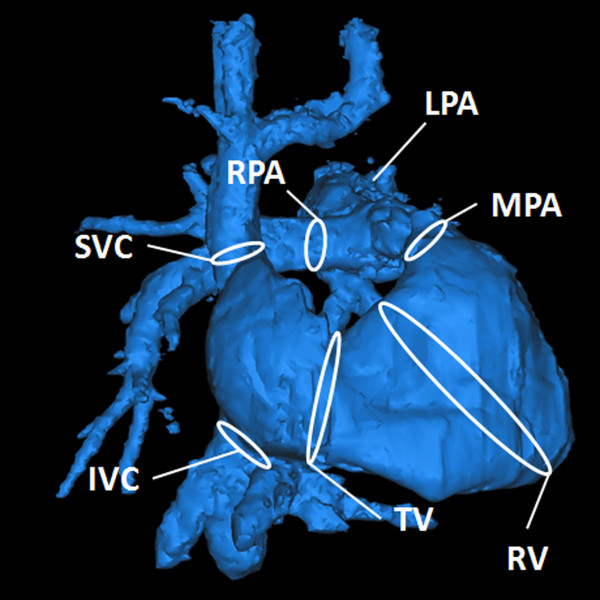
**15 year-old female with repaired Tetralogy of Fallot**. Surface shaded RV anatomy based on angiograms calculated from PC VIPR dataset. White circles indicate the locations of analysis planes used for emitting particle traces and streamlines for intracardiac flow analysis. The RV cutplane was placed from the apex of the curvature between the inlet and infundibulum to the apex of the RV.

During visualization with EnSight, a shaded surface display was created using the velocity-weighted MR angiogram. Analysis planes were manually placed orthogonal to the anticipated main flow direction (Figure 1) in the superior vena cava (SVC), inferior vena cava (IVC), right atrium (RA), RV, and the main, right, and left pulmonary arteries (MPA, RPA, and LPA, respectively). These 2D planes were used to emit 3D time-resolved particle traces and 3D streamlines. Particle traces are virtual massless particles that follow the acquired velocity vectors from one time step to another. Streamlines represent instantaneous paths that are tangent to the velocity vectors at a specific instant in time.

4D VM-CMR datasets were reviewed in a consensus reading with three experts in cardiovascular imaging (two radiologists and one cardiologist). Streamlines were reviewed at all discrete time steps of the cardiac cycle. Particle traces were viewed dynamically and in a step-wise manner. Both visualization options could be turned on and off for every analysis plane and were rotated or magnified for inspection in any chosen orientation such that readers could take full advantage of the entire 3D and time-resolved nature of the data.

Flow patterns in the SVC and IVC were characterized based on whether the majority of flow occurred during right ventricular systole or diastole. Normal flow patterns in the right heart have been previously described [7,14]. Based on these studies, characteristics used to describe normal and abnormal flow patterns are summarized in Table 3. Flow in the MPA, RPA, and LPA were characterized qualitatively as (A) normal with uniform, laminar-like flow field patterns or (B) abnormal with increased helical or vortical flow field features.

**Table 3:** Grading system for evaluating flow patterns in right heart chambers

RA	1	normal, single clockwise vortex^a^
	2	increased number of vortices
RV diastole	1	normal right-handed helix through TV
	2	increased number of vortices
RV systole	1	uniform, laminar flow toward RVOT
	2	non-uniform, random, or disorganized

RA = right atrium; RV = right ventricle

^a^when viewed from the patients' right with the sinus venosus posterior and tricuspid valve anterior.

Quantitative flow analysis was performed with previously described MatLab-based home built software (The MathWorks, USA) [15]. The main pulmonary arteries of all rTOF subjects and normal volunteers were segmented manually. Net flow, retrograde flow, peak systolic flow, time-to-peak flow, peak acceleration, resistance index and mean wall shear stress were measured in the MPA. The resistance index was calculated from the peak systolic and end-diastolic velocities (= (peak systolic velocity-end-diastolic velocity)/peak systolic velocity). Wall shear stress was calculated from the 3D velocity vector fields as described by Stalder, *et al*. [15].

### Statistical analysis

To compare the frequency of normal and abnormal flow patterns in the two groups, normal flow patterns were given a score of one and abnormal flow patterns were given a score of zero. Statistical analysis was performed using OpenStat (version 5/7/2010; Softonic, Barcelona, Spain). For each location analyzed, Fisher's exact test was used to analyze statistical significance of differences in flow patterns between the two groups. A non-parametric test (Kruskal-Wallis test) was used to analyze differences in quantitative parameters between groups, because of the small sample sizes in each group. The null hypothesis was rejected for P-values less than 0.05.

## Results

**Figure 2:**
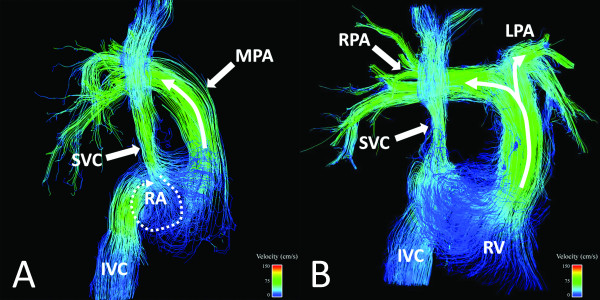
**21 year-old healthy female volunteer**. Streamline visualization in a right ventricular (RV) systolic phase. Right atrial (RA) filling primarily during systole, which is characterized by a single clockwise vortical flow pattern (A, curved dashed arrow). Simultaneously, smooth flow patterns are present in the main (MPA), right (RPA), and left (LPA) pulmonary arteries (B, curved solid arrows). Color-coding was achieved with respect the absolute acquired velocities.

**Figure 3:**
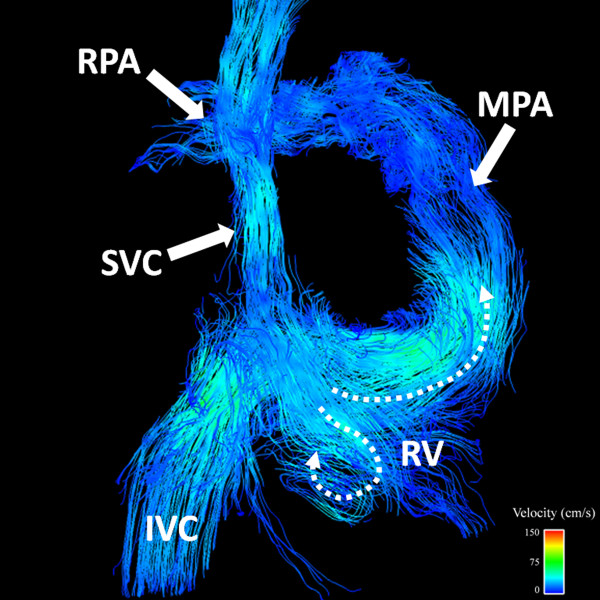
**28 year-old healthy male volunteer**. Streamline visualization during an early diastolic phase. In this typical example, two vortical flow patterns in the right ventricle (RV, dashed arrows), one directed toward the right ventricular outflow tract and one directed toward the inferior wall and tricuspid valve, can be observed. Color-coding was achieved with respect the absolute acquired velocities.

**Figure 4:**
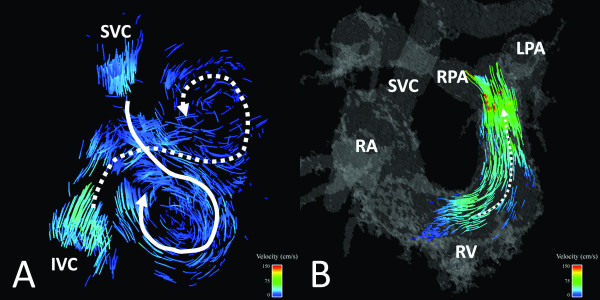
**9 year-old male with Tetralogy of Fallot status post Blalock-Taussig shunt and complete repair with transannular patch at 6 months of age**. (A) Snapshot of particle traces in the right atrium during right ventricular diastole reveales two distinct vortices. In addition to the normal clockwise vortex (solid curved arrow), a second vortex in the cephalad aspect of the right atrium was depicted (RA, dashed curved arrow). In (B) normal uniform flow in the RV and pulmonary artery outflow tract can be appreciated in a RV systolic time frame using particle traces visualization (dashed curved arrow). Color-coding was achieved with respect the absolute acquired velocities. SVC = superior vena cava; IVC = inferior vena cava; RPA = right pulmonary artery; LPA = left pulmonary artery.

**Figure 5:**
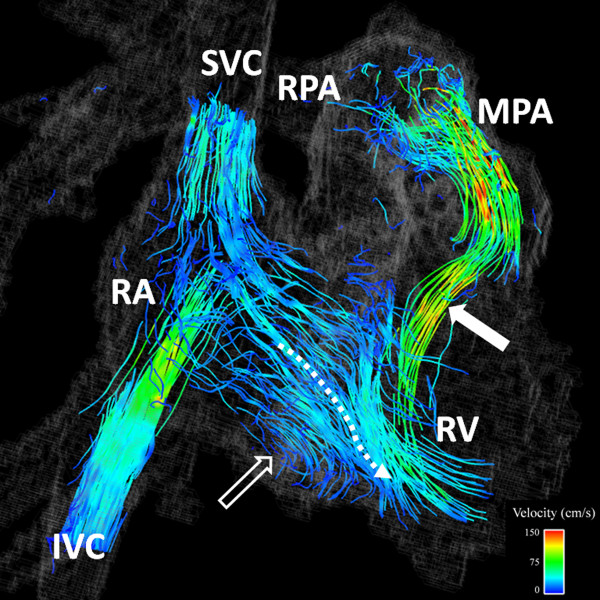
**17 year-old female with Tetralogy of Fallot repaired with transannular patch at 2 years of age**. Particle trace visualization during a right ventricular diastolic time frame demonstrates pulmonary regurgitation (closed arrow). The majority of the flow from the right atrium (RA) into the RV is directed abnormally toward the RV apex (curved dashed arrow) with a smaller vortex just beyond the tricuspid valve (open arrow). Color-coding was achieved with respect the absolute acquired velocities. SVC = superior vena cava; IVC = inferior vena cava; MPA = main pulmonary artery; RPA = right pulmonary artery.

**Figure 6:**
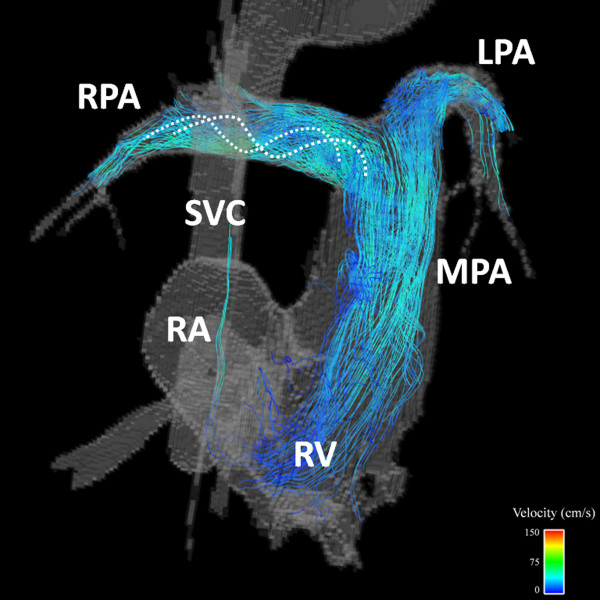
**38 year-old male with Tetralogy of Fallot status post complete repair and Waterston shunt takedown as infant**. Streamline visualization during a late systolic phase reveals increased helical flow patterns are present in the pulmonary arteries (curved dashed lines). SVC = superior vena cava; RA = right atrium; RV = right ventricle; MPA = main pulmonary artery; LPA = left pulmonary artery.

Based on clinically acquired 2D PC datasets, PS and PR were present in 7/11 and 8/11 subjects with rTOF, respectively. 4D VM-CMR with PC VIPR datasets were successfully acquired in all subjects. Results of the analysis of the flow patterns in right heart chambers are summarized in Table 4. Typical flow patterns in normal volunteers are shown in Figures 2 and 3 while typical flow patterns in subjects with rTOF are shown in Figures 4, 5, 6.

**Table 4:** Summary of flow patterns - number of subjects with normal flow patterns

Location	Normal volunteers (N = 10)^a^	Tetralogy of Fallot subjects (N = 11)^a^	P-value
SVC	10	3	<0.05
IVC	8	3	<0.05
RA	10	4	<0.05
RV diastole	10	1	<0.05
RV systole	10	9	0.48
MPA	10	5	<0.05
RPA	6	1	<0.05
LPA	10	1	<0.05

SVC = superior vena cava; IVC = inferior vena cava; RA = right atrium; RV = right ventricle; MPA = main pulmonary artery; RPA = right pulmonary artery; LPA = left pulmonary artery

^a^values reported are number subjects with normal flow, as detailed in the methods section and in Table 3.

### Qualitative analysis

#### SVC/IVC

Flow from the SVC into the RA was considered normal (greater flow during systole than diastole) in 10/10 normal volunteers and 3/11 subjects with rTOF. Flow from the IVC into the RA was described as normal in 8/10 normal volunteers and 3/11 subjects with TOF.

#### RA

Flow patterns in the RA in all healthy volunteers were considered normal and characterized by a single clockwise vortex (Figure 2A), when viewed from the patients' right with the sinus venosus posterior and the tricuspid valve anterior. In addition to abnormalities in timing of RA filling (diastolic filling greater than systolic filling), the flow in the RA was characterized by an increased number of vortices in 7/11 subjects with rTOF (Figure 4A).

#### RV

RV flow patterns in all healthy volunteers were considered normal, with a right-handed helix through the TV during diastole (Figure 3) and uniform, laminar flow toward the RVOT during systole (Figure 2B). Diastolic RV flow in rTOF subjects tended to be directed more toward the RV apex (Figure 5). This was associated with large PR jets also directed toward the RV apex. In addition, diastolic RV flow in rTOF subjects demonstrated an increase in the number of vortices, particularly in the subjects with larger RVEDV. RV systolic flow was normal in 9/11 rTOF subjects (Figure 4B).

#### PA

Normal volunteers exhibited normal uniform and laminar flow patterns in the MPA and LPA in all cases and in the RPA in 6/10 cases. In the four normal volunteer cases with abnormal flow in the RPA, increased helical flow was present. Subjects with repaired rTOF had normal flow patterns in the MPA in 5/11 cases and in the RPA and LPA in 1/11 cases. Increased helical and vortical flow patterns were present in the pulmonary arteries of the majority of rTOF cases (Figure 6).

The differences in flow patterns between normal volunteers and subjects with rTOF were statistically significant (P < 0.05) in all locations except for RV systole (P = 0.78).

### Quantitative analysis

Results of quantitative analysis of the MPA are summarized in Table 5. Statistically significant differences were present for pulmonary regurgitation, peak flow, time-to-peak flow, peak acceleration, resistance index and mean wall shear stress. Differences in net flow were not statistically significant.

**Table 5:** Summary of quantitative flow analysis

Parameter	rTOF	Volunteers	P-value
		
	MPA	MPA	
**Net flow (mL/cycle)**	40.8 ± 37.2	47.3 ± 26.2	0.514
**Retrograde flow (%)**	29.6 ± 21.4	7.4 ± 5.6	0.001
**Peak flow (mL/s)**	204.7 ± 126.5	395.8 ± 120.0	0.014
**Time-to-peak flow (ms)**	254.9 ± 121.1	104.2 ± 29.9	0.001
**Peak acceleration (L/s^2^)**	2.83 ± 2.25	6.77 ± 1.23	0.003
**Resistance index**	0.569 ± 0.275	1.053 ± 0.052	<0.001
**Wall shear stress (N/m^2^)**	0.668 ± 0.398	0.235 ± 0.028	0.004

Data are mean ± standard deviation.

rTOF = repaired Tetralogy of Fallot; MPA = main pulmonary artery

## Discussion

The findings of this study confirm that 4D VM-CMR with PC VIPR lends itself well to qualitative and quantitative analysis of the flow features of the entire right heart circulation. Similar to what has been recently reported by Geiger, *et al*. [16], we demonstrated similarities and differences in right heart flow patterns in normal healthy volunteers and in a broad age and size range of patients with palliated and repaired Tetralogy of Fallot. An important advantage of PC VIPR for evaluation of intra-cardiac flow patterns, relative to other approaches to 4D velocity flow mapping, is the volumetric chest coverage and isotropic spatial resolution (1.02-1.25 mm^3^) at clinically acceptable acquisition times on the order of 9-17 minutes. This provides a systematic approach for comprehensive hemodynamic evaluations of the heart and thoracic vasculature because all areas of potential interest are included within the imaging volume. By doing so, multi-planar reformations can be generated in all directions comprising perfectly co-registered morphologic and functional information without compromises in spatial resolution or additional scan times. Of note, the scan times for the PC VIPR acquisitions in this study (9-17 minutes) were similar to previously reported Cartesian approaches used for intra-cardiac flow pattern assessment with considerably worse (up to 9-times) spatial resolution [14].

Flow patterns in the RA and RV of normal volunteers were uniform and well organized, following patterns that have been described previously [7,14]. Of note, diastolic flow in the IVC was greater than systolic flow in two normal volunteers. Although the reasons for this cannot be determined definitively, these findings are not entirely unexpected. Appleton , *et al*. found that 10% of healthy subjects had greater diastolic than systolic forward velocity integrals in the SVC during at least one phase of respiration [17]. Flow in the vena cavae is known to change with differences in respiration and changes in intrathoracic and intraperitoneal pressures, with the IVC being more susceptible to these fluctuations than the SVC [18]. As expected, RA and RV flow patterns in patients with rTOF were dramatically different from those in normal volunteers. Diastolic flow in the RV was markedly abnormal in 10/11 rTOF patients, while systolic flow remained normal in 9/11 rTOF patients. The major inflow through the tricuspid valve in the repaired rTOF patients was directed toward the RV apex, usually as a result of concomitant pulmonary regurgitation. These findings agree with previous studies that evaluated impaired diastolic function in patients with repaired TOF [19-22]. In addition, impaired RV filling may help explain symptoms related to exercise these patients.

The alterations in filling of, and flow within, the RA are not unexpected in view of previous studies documenting alterations of RA function after repair of TOF [23,24]. These findings strongly suggest that the contribution of altered intracardiac flow patterns on morphodynamic coupling of diseases that affect the right heart circulation may have been underestimated thus far. We believe it reasonable to hypothesize that the extent of altered hemodynamics may precede morphological changes in the RV. 4D VM-CMR with comprehensive visualization of acquired flow fields may therefore have the potential to improve the characterization of the disease status. Clearly, such speculation cannot be based on the presented data and larger, longitudinal studies are warranted to further investigate the hemodynamics in RV failure. However, the results of this study demonstrate that PC VIPR is a promising potential diagnostic tool to achieve this goal.

The changes in flow patterns in the pulmonary arteries is concordant with the findings of Geiger, *et al*. who also reported the presence of abnormal vortices in the pulmonary arteries of patients with rTOF [16]. Although we did not measure the size of the pulmonary arteries, changes in MPA, RPA, and LPA size may contribute to the development of increased helices and vortices in dilated or post-stenotic segments. This phenomenon is commonly seen in aortic aneurysms [6,25,26]. Another factor in the development in vortices may be the increased pulmonary vascular resistance and elevated pulmonary arterial pressures. Reiter, *et al*. [27] reported that vortices develop in the MPA in patients with pulmonary arterial hypertension (PAH) secondary to flow separation between the boundary layer adjacent to the vessel wall, which is susceptible to the formation of vortical flow. The reduced acceleration time in the MPA in the rTOF subjects is also concordant with the findings of Reiter, *et al*. in subjects with PAH [27].

Although quantification of flow was not validated in this study, a benefit of using 4D VM-CMR techniques in complex CHD is that it is possible to *a posteriori *quantify flow volumes through any region of interest. This is particularly valuable in patients with complex congenital heart disease that frequently have multiple vessels through which flow quantification is required. To prospectively prescribe 2D velocity mapping acquisitions requires careful planning and a separate magnetic resonance angiography acquisition while the patient is on the scanner. Using 4D VM-CMR has the potential to simplify the acquisition of flow data and lead to decreased time for image acquisition.

Recently, 4D VM-CMR has been used to estimate wall shear stress *in vivo *[15,25,28,29]. Our observation of increased wall shear stress in the main PA of patients with rTOF is concordant with findings reported by E. Bedard, et al. on the histological abnormalities present in the pulmonary trunk of patients with TOF [30]. These authors reported an increased prevalence of medionecrosis, fibrosis, cyst-like formation, and abnormal elastic tissue configuration in the pulmonary trunks of patients with TOF. Presumably, these alterations in pulmonary trunk composition contribute to alterations in its mechanical properties. Although not the focus of this study, 4D VM-CMR could be use to quantify wall shear stress in the ascending aorta [15,25,28,29] as well to monitor for changes in the mechanical properties of the ascending aorta known to exist in patients with TOF [31]. Although still controversial, changes in wall shear stress have been implicated in the development and growth of aneurysms - with high wall shear stress associated with initiation [32] and low wall shear stress correlated with growth of [33] cerebral aneurysms. Analysis of the changes in the mechanical properties of the PA with 4D VM-CMR could potentially provide a means of further understanding PA-RV coupling leading to RV failure in patients with TOF as well as the changes that occur in aorta stiffness and aorta aneurysm development.

A major limitation of this study is that only a small number of rTOF subjects were examined and there was variability in types of repair that were performed. As a result, it is not possible to determine the relationship between the type of repair and its impact on RA and RV flow. The effects of type of repair will be the subject of subsequent investigation and could potentially provide insight into the relative advantages of different repairs on long term prognosis. Another potential drawback of this study is that the normal volunteers and patients were not age-matched, with normal volunteers having a greater mean age than the rTOF subjects, although it is unlikely that this would contribute to the relative differences in the observed alterations in right heart flow patterns. An additional limitation of this study is that we did not perform 4D VM-CMR with a Cartesian acquisition. Therefore, although we would assume that it is beneficial to have greater 3D spatial coverage with higher spatial resolution, it is not possible to confirm these theoretical benefits from the results of this study.

## Conclusions

In conclusion, this preliminary study demonstrates the feasibility of using 4D VM-CMR of the entire right heart circulation using PC VIPR. The flow patterns and quantitative flow parameters observed in repaired TOF patients were markedly different from that in normal healthy hearts. Using 4D VM-CMR to analyze flow related features may help to better understand the interactions between pulmonary regurgitation, tricuspid regurgitation, pulmonary artery stiffening and right heart function. Analysis of different types of TOF repair using 4D VM-CMR will be necessary to determine which quantitative parameters, if any, best predict outcomes in patients with repaired TOF.

## Competing interests

The authors declare that they have no competing interests.

## Authors' contributions

CJF conceived the original study and assisted with study design, data acquisition, analysis, and manuscript preparation.

SS assisted with analysis and manuscript preparation.

MLS assisted with study design, data acquisition, analysis, and manuscript preparation.

SBR assisted study design, data acquisition and manuscript preparation.

EN performed data analysis and assisted with manuscript preparation.

BRL performed data analysis and assisted with manuscript preparation.

OW assisted with study design, analysis, and manuscript preparation. AF assisted with study design, data acquisition, analysis, and manuscript preparation.

All authors read and approved the final manuscript.
